# Magnetic separation and concentration of Aβ 1–42 molecules dispersed at the threshold concentration for Alzheimer’s disease diagnosis in clinically-relevant volumes of sample

**DOI:** 10.1186/s12951-023-02095-8

**Published:** 2023-09-15

**Authors:** Alessandro Surpi, Mauro Murgia, Sonia López-Amoedo, Manuel A. González-Gómez, Yolanda Piñeiro, José Rivas, Valeria Perugini, Matteo Santin, Tomás Sobrino, Pierpaolo Greco, Francisco Campos, Valentin Alek Dediu

**Affiliations:** 1 Istituto per lo Studio dei Materiali Nanostrutturati (CNR-ISMN), Bologna, 40129 Italy; 2 Istituto per la Microelettronica e i Microsistemi, IMM-CNR, 40129 Bologna, Italy; 3 Center for Translational Neurophysiology (IIT), Italian Institute of Technology, Ferrara, 44121 Italy; 4 Translational Stroke Laboratory (TREAT), Clinical Neurosciences Research Laboratory (LINC) , Health Research Institute of Santiago de Compostela (IDIS), Santiago de Compostela, 15782 Spain; 5 NANOMAG Laboratory, Applied Physics Department, iMATUS Materials Institute, Universidade de Santiago de Compostela, Santiago de Compostela, 15782 Spain; 6 Centre for Regenerative Medicine and Devices, School of Pharmacy and Biomolecular Sciences, University of Brighton, Brighton, UK; 7 Centro de Investigación Biomédica en Red en Enfermedades Neurodegenerativas (CIBERNED), Instituto de Salud Carlos III, Madrid, 28029 Spain; 8 NeuroAging Group (NEURAL), Clinical Neurosciences Research Laboratory (LINC), Health Research Institute of Santiago de Compostela (IDIS), Santiago de Compostela, 15706 Spain; 9 Dipartimento di Neuroscienze e Riabilitazione, Università di Ferrara, Ferrara, 44121 Italy

**Keywords:** Alzheimer’s disease, Diagnosis, Magnetic nanoparticles, Molecular recognition

## Abstract

**Background:**

Alzheimer’s disease (AD) is the leading cause of dementia and loss of autonomy in the elderly, implying a progressive cognitive decline and limitation of social activities. The progressive aging of the population is expected to exacerbate this problem in the next decades. Therefore, there is an urgent need to develop quantitative diagnostic methodologies to assess the onset the disease and its progression especially in the initial phases.

**Results:**

Here we describe a novel technology to extract one of the most important molecular biomarkers of AD (Aβ_1−42_) from a clinically-relevant volume − 100 µl – therein dispersed in a range of concentrations critical for AD early diagnosis. We demonstrate that it is possible to immunocapture Aβ_1−42_ on 20 nm wide magnetic nanoparticles functionalized with hyperbranced KVLFF aptamers. Then, it is possible to transport them through microfluidic environments to a detection system where virtually all (~ 90%) the Aβ_1−42_ molecules are concentrated in a dense plug of ca.50 nl. The technology is based on magnetic actuation by permanent magnets, specifically designed to generate high gradient magnetic fields. These fields, applied through submillimeter-wide channels, can concentrate, and confine magnetic nanoparticles (MNPs) into a droplet with an optimized shape that maximizes the probability of capturing highly diluted molecular biomarkers. These advancements are expected to provide efficient protocols for the concentration and manipulation of molecular biomarkers from clinical samples, enhancing the accuracy and the sensitivity of diagnostic technologies.

**Conclusions:**

This easy to automate technology allows an efficient separation of AD molecular biomarkers from volumes of biological solutions complying with the current clinical protocols and, ultimately, leads to accurate measurements of biomarkers. The technology paves a new way for a quantitative AD diagnosis at the earliest stage and it is also adaptable for the biomarker analysis of other pathologies.

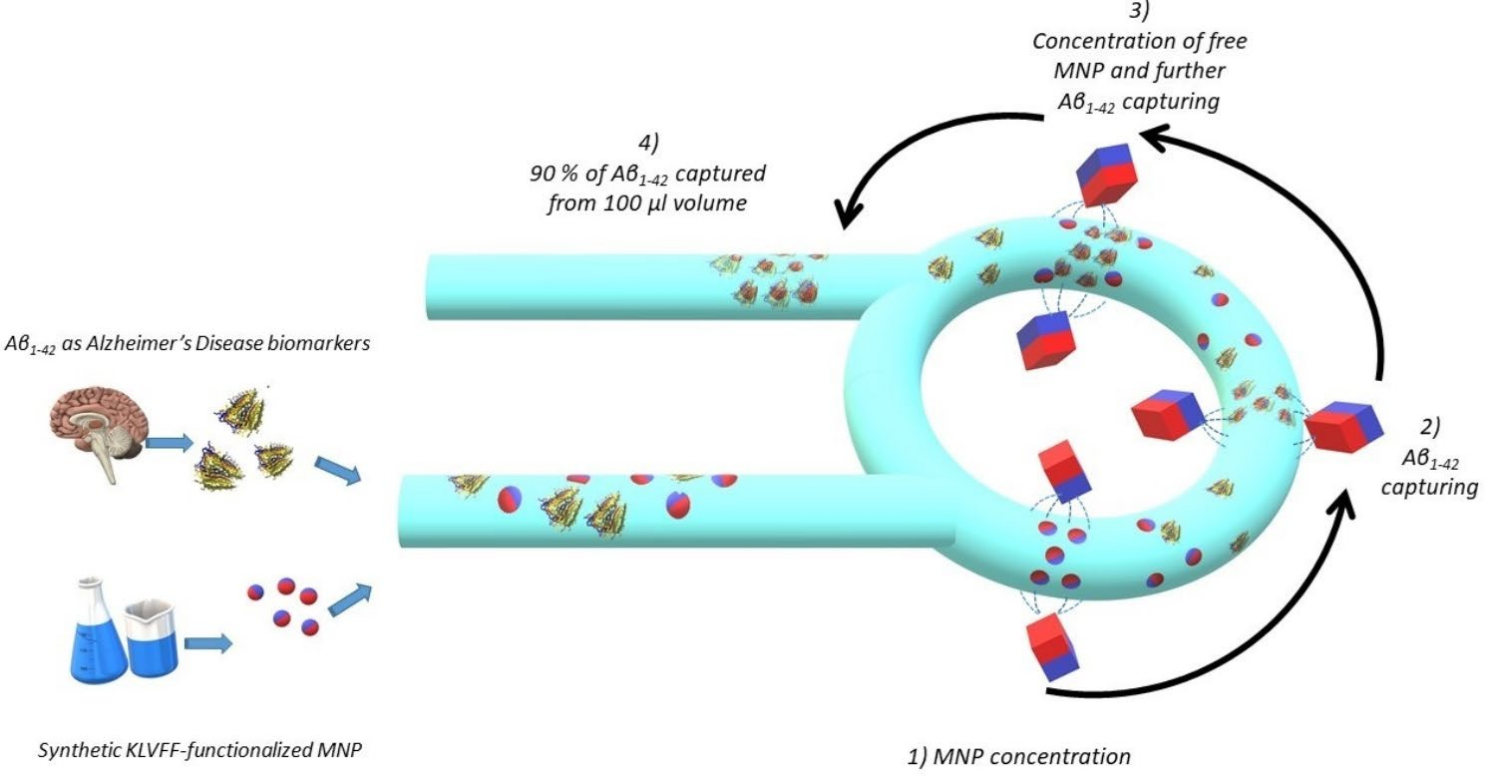

## Background

Alzheimer’s disease (AD) is a progressive neurodegenerative disorder with high epidemiological relevance and significant social impact. Although, the molecular causes of AD are not well-defined, it is widely accepted that presence of amyloid plaques in the brain, caused by the aggregation of Aβ_1−42_ and Aβ_1−40_ in insoluble oligomers and fibrils, represents one of its main pathological hallmarks. These plaques trigger pathological processes leading to neuronal damage and subsequent cognitive impairment [1–3]. An inverse relation exists between the presence of amyloid plaques in the brain - as imaged by *in-vivo* positron emission tomography - and concentration of Aβ_1−42_ in cerebral spinal fluid (CSF) even in early patients [4]. It has been also demonstrated that analysis of CSF Aβ_1−42_ significantly increases the diagnostic accuracy in clinically uncertain cases [5]. Because of that, in 2014, the International Working Group for New Research Criteria for the Diagnosis of Alzheimer’s disease (IWG) proposed a revised version of the diagnostic criteria for AD where CSF levels of Aβ_1−42_ should be considered as pathophysiological markers at any stage of the disease, preclinical states included [6]. Therefore, sensitive, specific, and robust methods to quantify Aβ_1−42_ concentrations in body fluids are central in AD research, drug development and clinical management [6, 7]. They are also critically important for early diagnosis as new drugs have recently shown in phase III trials to slow cognitive and functional decline in patients with early AD [8].

Recent technological advances have made it possible to measure Aβ_1−42_ concentration in CSF using automated assays [9]; yet, novel technical tools are needed to enhance the sensitivity to the level required for fast standardized brain amyloidosis monitoring during the clinical practice [10]. Additionally, measurements of Aβ_1−42_ concentration are critically sensitive to pre-analytical and analytical biases [6, 11, 12]. Fully automated instruments (from biological fluid handling to detection) could therefore significantly improve the current state-of-the-art.

Microfluidic devices are of great interest for a quantitative measure of AD biomarkers [13] and the use of magnetic nanoparticles (MNPs) can facilitate their miniaturization into lab-on-a-chip platforms as proper functionalization can enable MNPs to selectively bind to the target biomolecule. Subsequently, external magnetic fields can be used for manipulation and detection of the MNP-bound biomolecule [14]. Magnetic actuation can also solve the critical issue in microfluidics: mixing and processing of fluids become inefficient at small scales because of the dominance of capillary and viscous forces [15].

MNPs are used in biomedicine to bind cancer biomarker proteins [16], DNA/RNA sequences [17, 18], bacteria [19], eukaryotic cells [20, 21] or extracellular vesicles [22] for sensing and separation purposes even from complex biological matrices. Their remote control in microchannels have been demonstrated by using rotating magnetic systems [23]. Advanced lithography has also allowed the fabrication of complex patterned surfaces on which the motion of MNPs (and the biomolecules bound to them) can be finely controlled: thin films patterned into arrays of micro-sized soft magnetic structures [24] or electromagnets [25]. Magnetic domain walls in continuous ferromagnetic thin films can also be used to this aim [26].

However, most of those technologies failed to progress into a real-world clinical tool as they require cumbersome external devices and/or elaborate chip designs. As a result, there is an increasing interest in the use of permanent magnets as a simple and effective approach for MNP manipulation [27–29].

Here, we report the development of an experimental device for the capture and concentration of Aβ_1−42_ amyloids in biological fluids, based on magnetic actuation of superparamagnetic MNPs by permanent magnets arranged as in [30]. In comparison with similar devices reported in ref. [27, 28], the magnet arrangement here employed concentrates the MNPs in a clepsydra-shaped configuration (CSC). Its shape is optimized to capture much-diluted molecules and maximizes the MNPs active area for molecular binding as it spans across the entire capillary section while not forming large dense concentrations where most of the MNPs are buried in “*dead layers”*. We experimentally demonstrate that the device here presented can immune-capture Aβ_1−42_ molecules uniformly distributed in clinically relevant volumes of fluid – 100 µl – in a range of concentrations clinically relevant for AD early diagnosis [31]. It can also concentrate virtually (90%) all the molecules dispersed in the macroscopic volume of fluid into a nanoliter-sized plug and reliably transport them to a defined point where a sensor can be placed.

## Methods

### Experimental device

**Fig. 1:**
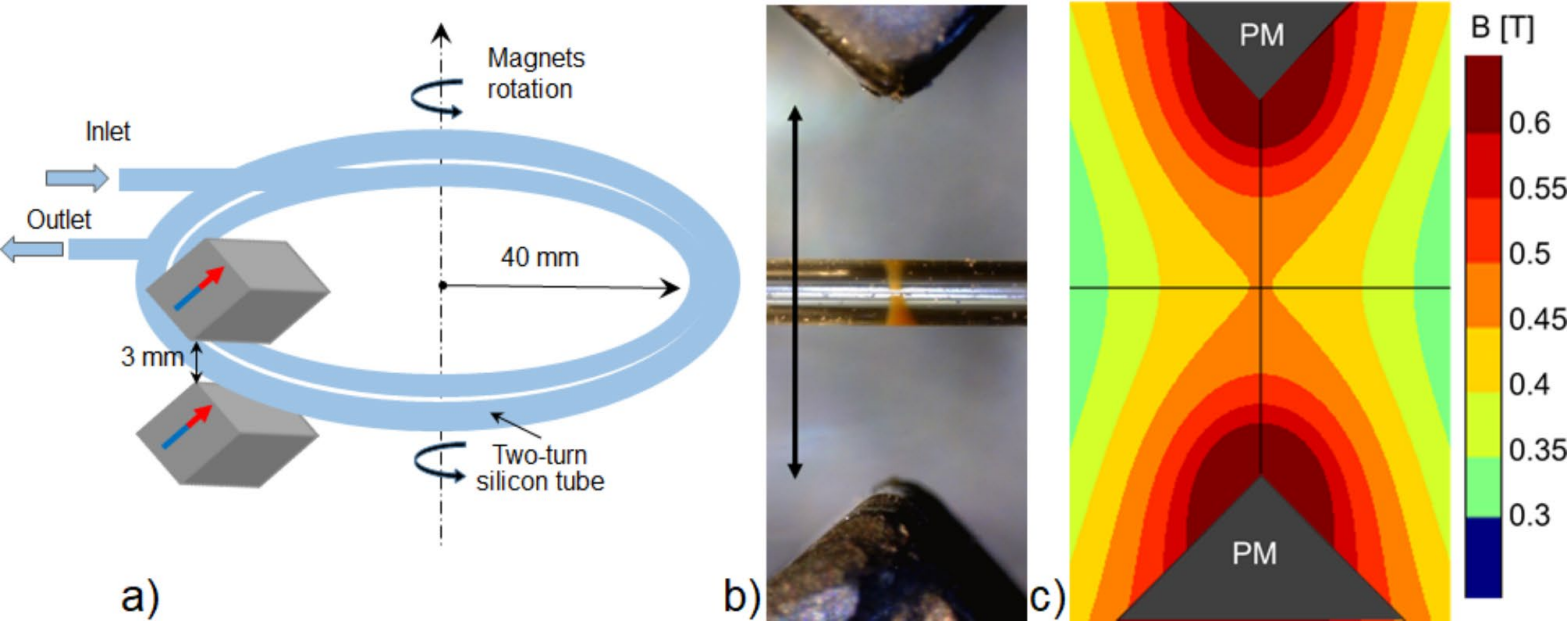
**a** Configuration of the magnets in the experimental device and its working principle. A couple of magnets are symmetrically placed on the sides of the silicone tube and are moved along following the curvilinear geometry. **b** Experimental realization of MNP magnetic confinement in a capillary having the same diameter as the internal lumen of the silicone tube used in the experiments. The MNP plug is contained in a 250 μm long section of a 500 μm wide capillary and thus has a volume of 50 nl. Bar is 3 mm long corresponding to the edge-to-edge distance between magnets. The image is oriented as in **c.** For imaging purposes, the magnetically confined MNP plug is shown in a completely transparent quartz capillary suspended between the magnets. Reprinted from [30]. **c** FEM-based simulation of the magnetic field generated by the magnets used in the experimental device by the permanent magnets (PM).

The device is composed of a fork-like magnet holder that keeps two 20 × 5 × 5 mm^3^ NdFeB magnets with 1.3 T as remanence field (Supermagnete GmbH, Gottmadingen, Germany), symmetrically placed at a fixed edge-to-edge distance of 3 mm. A rotatory motor moves the magnets holder, via a central shaft, along a 0.9 mm wide (internal lumen diameter is 500 μm) medical-grade silicone tube (Silastic®, Dow Corning, Midland, Michigan, USA) wrapped in a two-turn spiral (radius = 40 mm). The longest dimension of the magnets is kept orthogonal to the tube’s direction during the entire rotation. A polycarbonate housing keeps the flexible silicone tube at the midpoint between the magnets and allows the tube endings to be connected on one side to the peristaltic pump feeding the system and on the other to an outlet. The configuration of the magnets and their positioning with respect to the tube is sketched in Fig. 1a. Briefly, the magnets generate a magnetic field with a clepsydra-like shape: it peaks between the magnets along the (local) direction of the capillary but, in the perpendicular direction, has a broad minimum centred at the midpoint. Maxima are located on the magnets (Fig. 1c). Size and position of the tube play a crucial role in the magnetic confinement of the MNPs. Indeed, a 500 μm-wide tube placed at the midpoint between the magnets physically constrains the MNPs in a 250 μm-long slice where the field is fairly constant. There inside the MNPs are confined in a clepsydra-shaped configuration (CSC) spanning across the entire tube’s section (Fig. 1b) resembling the magnetic field distribution. (Fig. 1c). MNPs remain confined in a stable CSC because of the high field gradients along the longitudinal direction (to the tube) attracting MNPs between the magnets. Instead, along the transversal direction, the field gradients are low at the midpoint between the magnets, and this allows the MNPs to disperse across the entire tube’s section. Were the tube wider or displaced from the centre (where the gradients are steeper along the perpendicular direction), the MNPs would be attracted towards higher field regions and ultimately coalesce in dense blobs. A more detailed description of the magnetic configuration has been recently published in [30], where both the static confinement and the possibility to move the clepsydra along microfluidic channels were investigated. Crucially for the application here presented, the CSC remains stable when the magnets are moved along the two-turn spiral tube at a speed of 40 μm/sec, in agreement with previous publication [30].

### Synthesis and characterization of MNPs

The synthesis of MNPs functionalized with the Aβ_16−20_ residue Lys-Leu-Val-Phe-Phe (KLVFF) involves two subsequent steps: preparation of core-shell Fe_3_O_4_@SiO_2_ nanoparticles and surface modification with hyperbranched poly(epsilon-Lysine) dendrons exposing KLVFF sequences at their uppermost branching generation.

### Preparation of core-shell nanoparticles

#### Synthesis of silica-coated magnetite nanoparticles (Fe_3_O_4_@SiO_2_ MNPs)

All analytical grade reagents were obtained from MERCK (Saint Louis, MO, USA) and used as received without any further purification. Fe_3_O_4_@OA MNPs were obtained by co-precipitation method, following the procedure described in ref. [32] with some modifications. For this, iron(III) chloride hexahydrate (FeCl_3_·6H_2_O, 45 mmol) and iron(II) sulfate heptahydrate (FeSO_4_·7H2O, 30 mmol) were dissolved in 100 ml of 10 mmol hydrochloric acid (HCl) aqueous solution with mechanical stirring. The mixture was heated up to 60 °C, then ammonium hydroxide (NH_3_ aq, 770 mmol) and oleic acid (OA, 7.1 mmol) were added, and the reaction was carried out for 1 h. After that, the obtained MNPs were transferred to a beaker and placed on a hot plate at 100 °C to allow flocculation. The precipitate containing Fe_3_O_4_@OA MNPs was separated from the reaction medium by a magnetic field and washed three times with Milli-Q water (Millipore®, Burlington, MA, USA). Finally, Fe_3_O_4_@OA MNPs were re-dispersed in cyclohexane (CHX) and the remaining water was completely removed from the organic phase by using a decantation funnel. The total solid content was determined by thermogravimetric analysis (TGA, Perkin Elmer model 7, Waltham, MA, USA): W_mag_ = 4.2% by weight.

**Fig. 2:**
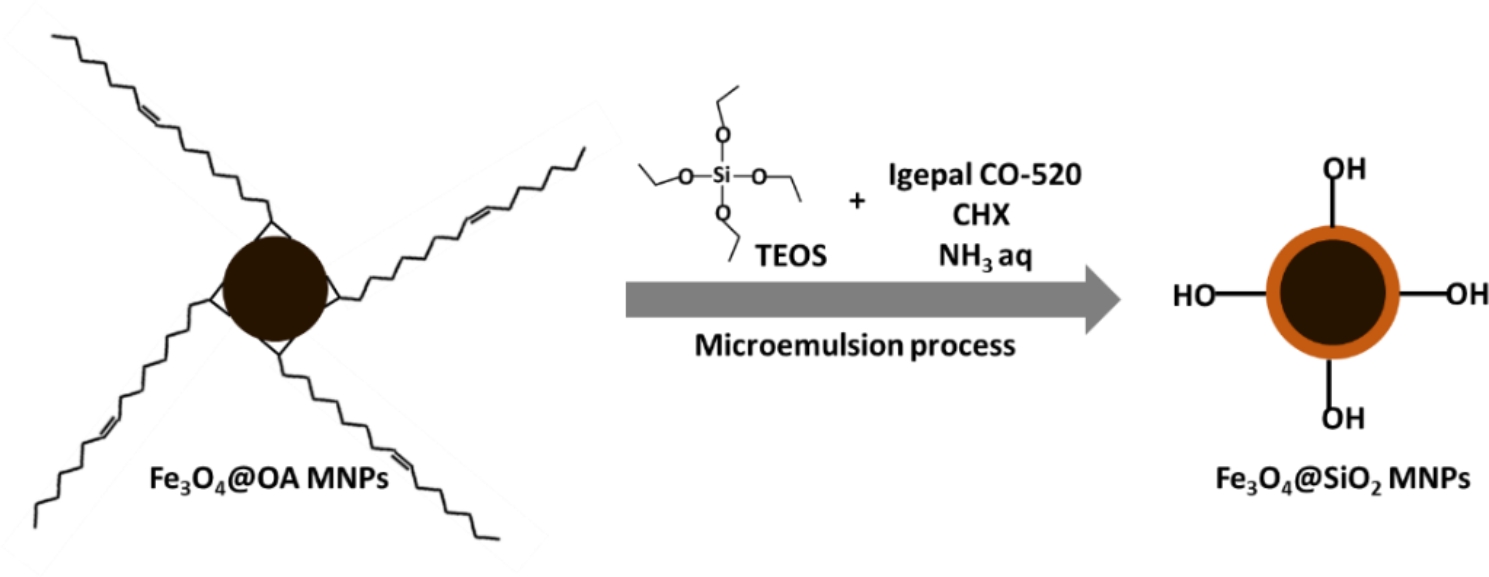
Sketch of the methods for Fe_3_O_4_@SiO_2_ MNPs synthesis as detailed in the text

Starting from Fe_3_O_4_@OA MNPs, core-shell Fe_3_O_4_@SiO_2_ MNPs were prepared according to a water-in-cyclohexane reverse microemulsion process as reported in ref. [33] with some modifications. Briefly, 77 mg of Fe_3_O_4_ MNP@OA dispersed in CHX were added to a mixture of Igepal CO-520 (19.5 mmol) and CHX (2 mol). The mixture was stirred at 350 rpm at room temperature for 30 min. Then, NH_3_ aq (15 mmol) and tetraethyl orthosilicate (TEOS, 10.7 mmol) were added, the mixture was covered with aluminium foil, and stirred for 16 h at room temperature. The obtained Fe_3_O_4_@SiO_2_ MNPs were washed 4 times using 2-propanol (IPA). For each wash, the MNPs were retained with a magnet and the supernatant was removed. Finally, the MNPs were washed twice with Milli-Q water and centrifuged at 9000 rpm for 10 min. The silica coated magnetite nanoparticles were redispersed in Milli-Q water. The total solids content was determined by TGA: W_mag_ = 1.33% by weight. The synthesis process is visually described in Fig. 2.

#### Structural characterization

**Fig. 3:**
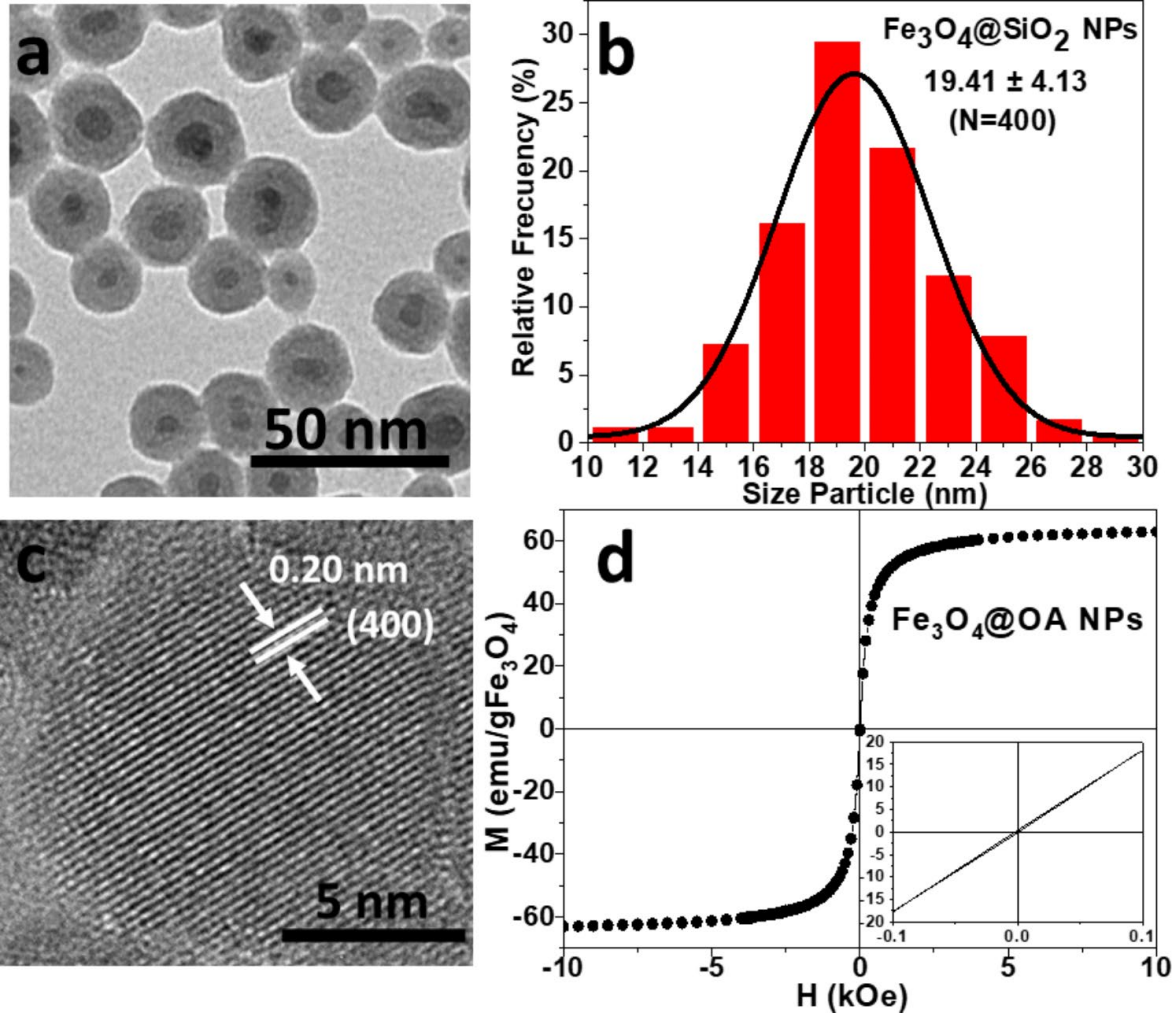
Morphological, structural, and magnetic characterization of Fe_3_O_4_@SiO_2_ MNPs: **a** TEM image, **b** particle size distribution, **c** HRTEM image of Fe_3_O_4_@OA MNPs (magnetic core) showing lattice fringes with d-spacing of 0.20 nm, which is a characteristic of the (400) magnetite structure, and **d** hysteresis loop at room temperature

The MNPs were characterized by different techniques to assess their physical and chemical properties. Morphology was characterized by transmission electron microscopy (TEM) using a JEOL JEM-1011 microscope (JEOL, Tokyo, Japan) operating at 100 KV. In addition, the structure and properties of the MNPs were analyzed at the atomic scale in a selected area by electron diffraction (SAED) with High Resolution TEM LIBRA 200FE (HRTEM, Carl Zeiss NTS GmbH, Oberkochen, Germany) operating at 200 KV. TEM micrograph of Fe_3_O_4_@SiO_2_ MNPs (Fig. 3a) shows a spherical morphology with a diameter around 20 nm, where magnetite cores (dark contrast) are embedded inside the silica shell (bright contrast). It can be appreciated that the reverse microemulsion method allowed the development of an excellent silica coating and a very narrow size distribution as shown in Fig. 3b. The HRTEM image (Fig. 3c) confirms the crystalline nature of the magnetite nanoparticles. A typical magnetization curve for NP is shown in Fig. 3d.

**Fig. 4:**
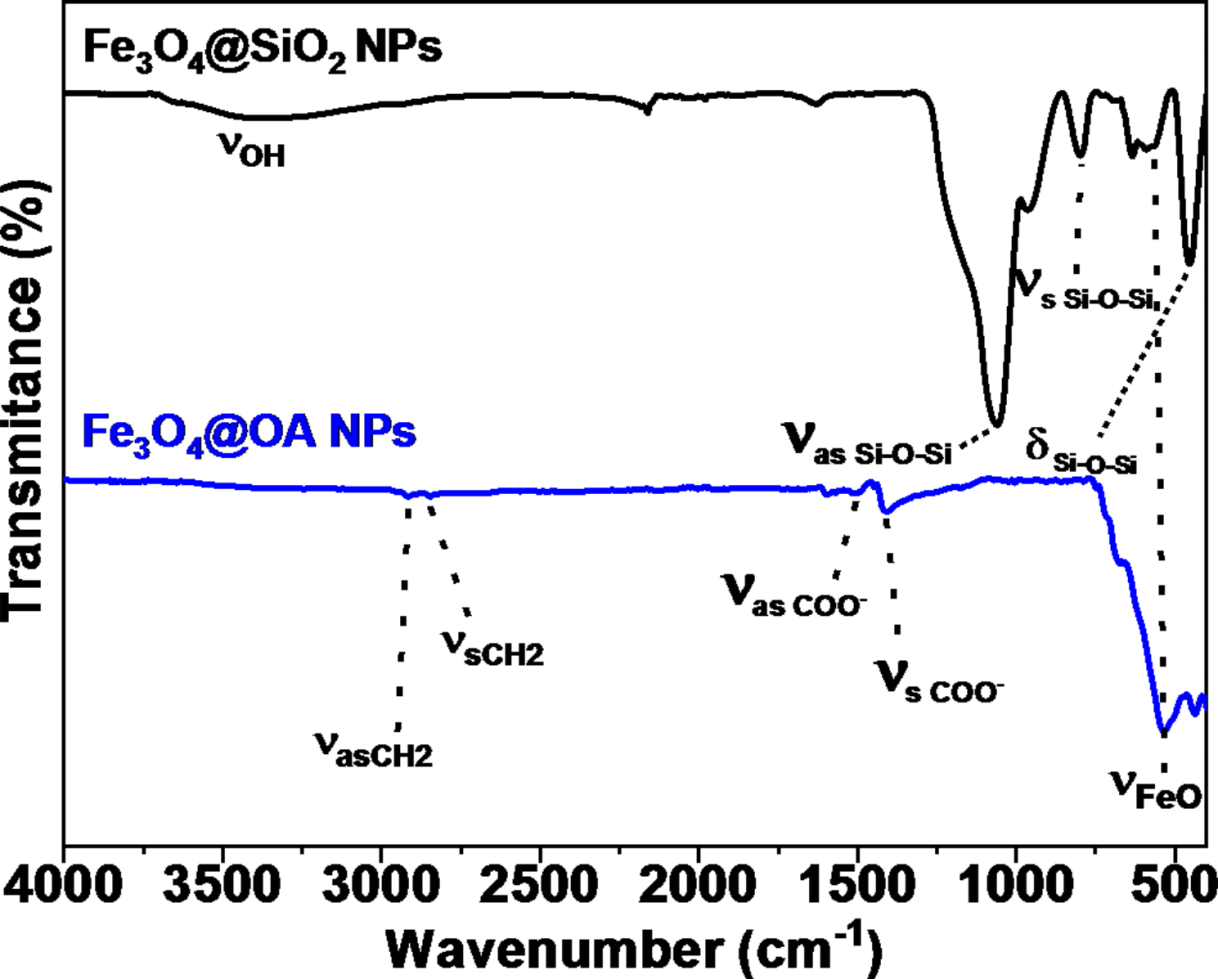
FTIR spectrum of the magnetite nanoparticles (blue pattern) and silica-coated magnetite nanoparticles (black pattern), with the characteristic bands as evidence

The sample shows negligible coercive fields (H_C_=3.5 Oe) and remanence (M_R_=0.6 emu/g), corresponding to a superparamagnetic (SP) behaviour. The saturation magnetization of 63 emu/g_Fe3O4_ is comparable with the highest values reported in literature for iron oxide SP-MNP [34]. Surface functional groups of dried MNPs were analyzed by Fourier Transform Infrared (FTIR) Spectroscopy with a Thermo Nicolet Nexus spectrometer (Thermo Fisher Scientific, Madrid, Spain) using the attenuated total reflectance (ATR) in the range of 4000 − 400 cm^− 1^ and are shown in Fig. 4. Silica coating can be confirmed by the appearance of three peaks at 1069, 452 and 792 cm^− 1^, corresponding to the stretching modes of Si-O-Si (asymmetric and symmetric) and the scissoring vibration of Si-O-Si, respectively [35]. The hydrodynamic radius of 30 nm was measured for these MNPs in aqueous solution via Dynamic Light Scattering by a Malvern Nano ZS (Malvern Instruments, Malvern, UK).

### Surface modification with hyperbranced KVLFF

#### Synthesis of branched KLVFF aptamers

Molecular recognition of the selected biomarker is ensured by MNP surface functionalization with short peptides having binding affinity to specific regions of the Aβ_1−42_ and chemical stability [36]. Among them, the sequence KLVFF has been identified as one of the most effective one as it specifically binds to the homologous regions of Aβ_1−42_ [37].

Branched poly(epsilon-lysine) peptides - starting with an Arginine (R) molecule followed by the branching in 3 generations of poly(epsilon-lysine), and exposing the KLVFF aptamer sequence at each of the 16 uppermost molecular branches [RGen3K(KLVFF)_16_] - were assembled using a common 9-fluorenylmethoxycarbonyl (Fmoc) solid phase peptide synthesis (SPPS) by a microwave synthetiser (Biotage ® Initiator, Ystrad Mynach UK). A linear aptamer including a spacer of two Glycine molecules was also synthesised and later used as control in the MNP surface functionalisation to discriminate any negative effect (e.g. steric hindrance) that the larger branched aptamer has in the process of coupling to the nanoparticles.

Initially, 0.5 g of Tenta Gel S NH_2_ resin (Iris Biotech GmbH, Marktredwitz, Germany) was swollen with 5 ml dimethylformamide (DMF) (Fisher Scientific, Loughborough, UK), for 15 minutes. After intensive washes with 3 ml of DMF, the resin was coupled to the C-gorup of an amide linker, Fmoc Rink Amide Linker (Merck KGaA, Darmstadt, Germany), sonicated with 3 ml of DMF containing 0.4 mmol O-Benzotriazole-N,N,N’,N’-tetramethyl-uronium-hexafluoro-phosphate (HBTU) (Novabiochem, London, UK) and 0.8 mmol N,N-Diisopropylethylamine (DIPEA) (Merck KGaA, Darmstadt, Germany) used as activation agents. Reaction mixture was performed at 50 °C and 50 W for 6 min. The linker was then washed three times with DMF and its Fmoc-group was removed using 20% v/v piperidine (Merck KGaA, Darmstadt, Germany) in DMF following two deprotection steps at room temperature for 4 min/each. The aptamers were again washed three times in 5 ml of DMF while revealing new N-terminal amine that supported the assembly of an ordinate series of Fmoc-amino acids. After all amino acids were added by series of coupling and deprotection steps, all aptamers were allowed to stand for 30 min for final deprotection under stirring at 900 rpm and high absorption and transferred to a 10 mL fritted syringe through a series of washes with 40 ml dichloromethane, methanol and diethylether (Fisher Scientific, Loughborough, UK). Then, they were dried and weighed prior to be cleaved from the resin.

The final branched macromolecules were isolated from resin using a specific cleavage cocktail: 95% v/v trifluoroacetic acid, (TFA) (Fisher Scientific, Loughborough, UK) 2.5% v/v deionized water and 2.5% v/v trisopropylsilane (TIPS) (Merck KGaA, Darmstadt, Germany). After three hours incubation, each aptamer solution was passed down a Pasteur pipette filled with 1 cm of a glass wool and the crude aptamers were collected in a tube containing 20 ml of chilled diethylether (Fisher Scientific, Loughborough, UK) and centrifuged by a Denley BS400 centrifuge (Denley Instruments, Cambridge, UK) for three times at 3500 rpm for 5 min. Afterwards, aptamers were freeze dried using a Christ Alpha 2–4 LSC freeze-drier (SciQuip LTD, Wem, UK), dissolved in pure methanol and filtered through a syringe filter with a pore diameter of 0.25 μm (GE Healthcare, Amersham, UK) prior to their characterization.

#### Chemical coupling of Rgen3K(KLVFF)_16_ and linear GG-KLVFF on to Fe_3_O_4_@SiO_2_ MNPs surface

200 mg of MNPs of Fe_3_O_4_@SiO_2_ MNPs were taken from a 10 mg/ml concentration solution. The MNPs were centrifuged three times at 3500 rpm for 5 min, then the supernatant was removed and the MNP were left to air-dry for 48 h. 2 mg of Fe_3_O_4_@SiO_2_ MNPs were weighed and underwent a mild treatment with 3.0% (v/v) hydrogen peroxide to induce the formation of free hydroxyl radicals from the oxygen groups of silica. This consisted in a quick sonication in the hydrogen peroxide solution followed by 15 min incubation under orbital shaking. The Fe_3_O_4_@SiO_2_ MNPs were resuspended at a final concentration of 1 mg/ml in 2 ml of 0.1 mol MES buffer (pH 6.5) containing 0.2 mmol 1-Ethyl-3-(3-dimethylamino-propyl)carbodiimide (EDC) (Merck KGaA, Darmstadt, Germany) and 0.05 mmol N-Hydroxy-succinimide (NHS) (Merck KGaA, Darmstadt, Germany) to derivatise the hydroxyl groups into amino groups. The Fe_3_O_4_@SiO_2_ MNPs were rapidly sonicated and incubated with on an orbital shaker for 2 h at room temperature. The incubation medium was removed and the Fe_3_O_4_@SiO_2_ MNPs washed three times with deionised water by spinning them down at 3500 rpm for 5 min and finally conjugated with 50 ml solution of either aptamer solution (10 mg/ml) for 2 h at room temperature to promote the formation of peptide bonds of the aptamer with the derivatised Fe_3_O_4_@SiO_2_ MNPs surface. The MNPs were washed with deionised water three times and left to air-dry overnight. Prior to experiments of Aβ_1− 42_ capturing, control SiO2@MNP and RGen3K(KLVFF)_16_-functionalised MNPs were resuspended in Nanosperse® (Tissue Click Ltd, Brighton and Hove, UK), a biocompatible nanoparticle aqueous dispersant medium.

#### Material characterization

**Fig. 5:**
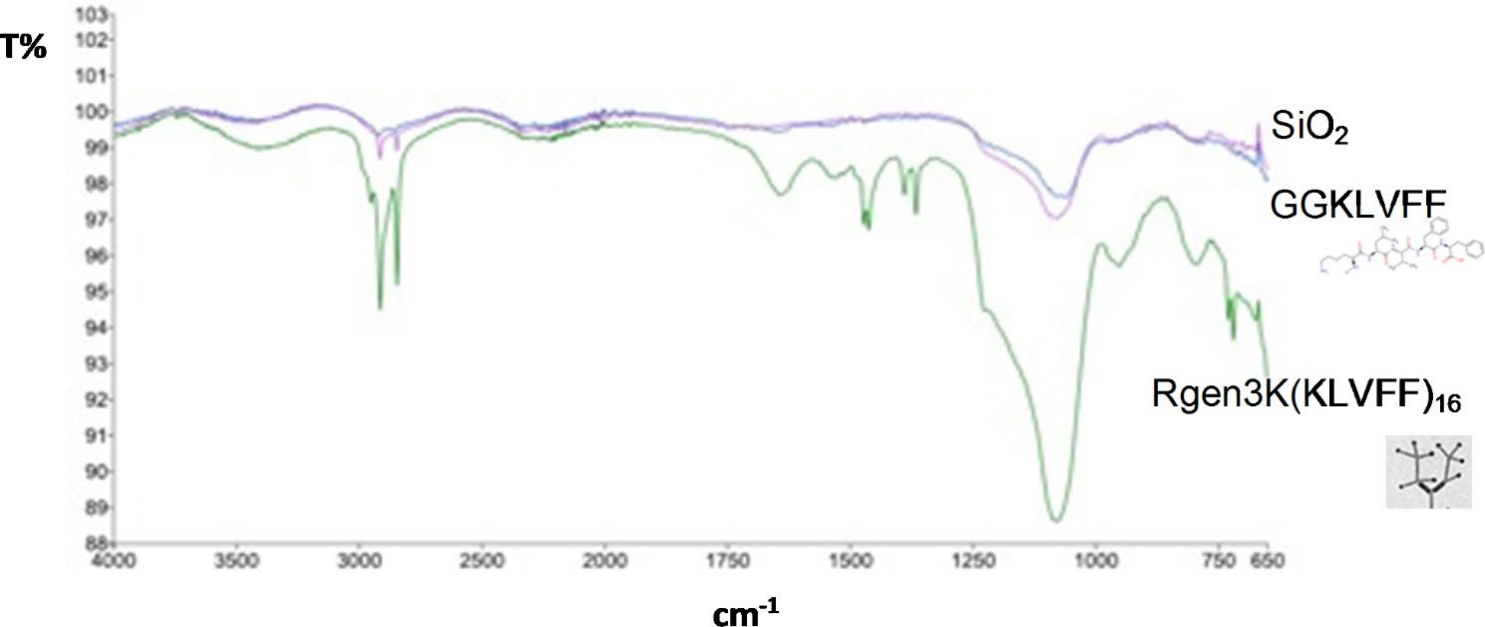
FTIR of the SiO_2_@MNP functionalised with a GG-KLVFF and RGen3K(KLVFF)_16_ branched aptamer

The pure solid-phase synthesis of branched KLVFF aptamers was demonstrated as a single peak in high-performance liquid chromatography (HPLC) spectra and FTIR indicated the successful functionalization of MNP with the branched aptamer (Fig. 5). As a control, MNP surface functionalization was also performed with single KLVFF sequence to which a Glycine-Glycine spacing arm was added instead of the branched poly(epsilon-Lysine). The emergence of peaks in the typical region of primary and secondary amines are clearly visible on MNPs functionalized with the hyperbranched aptamers. A hydrodynamic radius of 300 nm was measured for the KLVFF-functionalized MNPs via Dynamic Light Scattering by a Malvern Nano ZS (Malvern Instruments, Malvern, UK).

## Results and discussion

### Controlled transport of MNPs

**Fig. 6:**
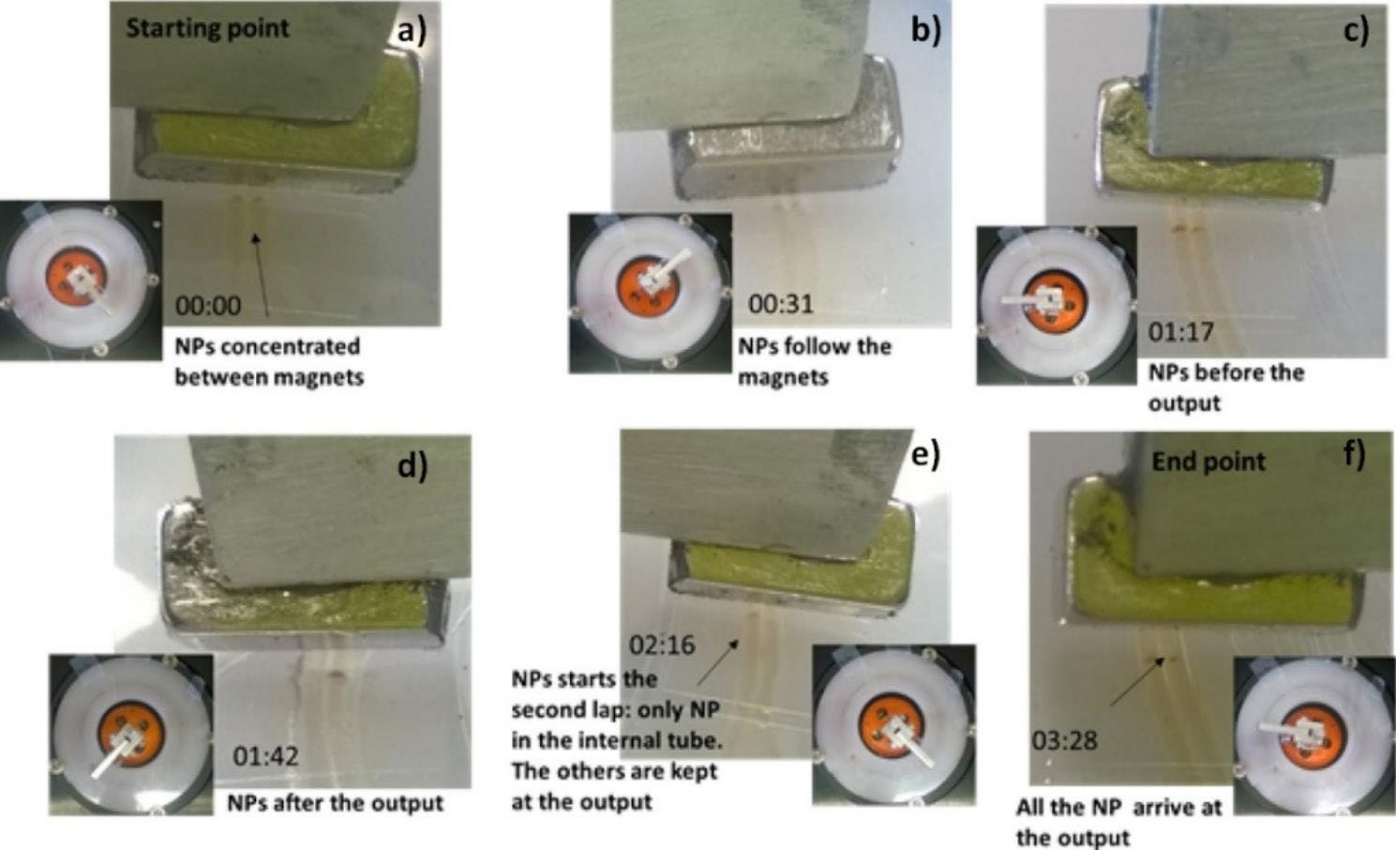
Controlled transport of MNPs. Time is given in hr:min format

In the device here described, the separation, concentration, and manipulation of Aβ_1− 42_ molecules is based on magnetically-controlled transport of MNPs through the two-turn spiral. It enables the concentration of MNPs into a dense CSC plug between the magnets from the nearby section of the silicone tube, the subsequent accumulation of more MNPs into the same plug as the magnets move along the spiral and, finally, the delivery of all the MNPs initially dispersed in the solution at the device’s outlet as shown in Fig. 6 (in the following the different phases of the process are indicated by the letter in the relative image of Fig. 6**)**. The specific functionalization of the MNPs allows the capture of Aβ_1− 42_ molecules on their surface and ultimately their manipulation. At the beginning of the experiment, the KVLFF-functionalized MNPs were suspended in a Aβ_1− 42_ Lumipulse® calibration solution (Fujirebio, Gent, Belgium) of known concentration and diluted in buffer with chemical stabilizers and, as preservative, 0.1% 2-pyridinol-1-oxide to avoid amyloids degradation. Then, 100 µl of this suspension (roughly the two-turn spiral volume) was injected by suction through the inlet using an infusion pump (Harvard-Apparatus PhD-ultra, Holliston, MA, USA) with a flow rate of 500 µl/min. The injection was done with the magnets at the inlet. After the capillary was filled, we waited roughly 10 min to concentrate MNPs into a CSC between magnets **(a)**. Then, the magnets started running from the initial position at the inlet **(b)** and rotated for 3.5 h covering the two-loop tubing at a speed of 40 μm/sec. This value assures that the CSC remains stable while moving along the capillary as the chosen speed sits in the middle of the “*stability window*” defined in [30]. Because the silicone tube is transparent, it is possible to visually follow the progression of the MNP droplet following the magnets. The MNPs are progressively concentrated between the magnets as they travel along the two-turn spiral. At the beginning, MNPs are concentrated in both the spiral turn **(c)**. As the magnets travel to the output **(d)**, the MNPs in the external turn reach the junction with the output and there they remain while the ones in the internal turn keep on following the magnets for a second round (passing from the internal to the external turn) **(e)**. Finally, when the magnets reach for the second time the junction all MNPs were collected in a dense plug located at the output **(f)**. In this way, we ensure that the complete sample was systematically scanned by the KVLFF-functionalized MNPs for the targeted Aβ_1− 42_ molecules. As detailed in [30], the dynamics of the MNPs magnetically trapped in this device is dictated by the magnetic force. Indeed, once a MNP is attracted in the region between the magnets, the magnetic energy dominates over the thermal energy responsible for the destabilizing effects of the Brownian motion. This assures an efficient manipulation of MNPs independently of their hydrodynamic radius, such as bare MNPs, KLVFF-functionalized MNPs and finally KLVFF-functionalized MNPs bond with Aβ_1− 42_ molecules.

### Assessing Aβ_1−42_- KVLFF functionalized MNPs binding probability

The capture, separation, and transport of Aβ_1−42_ by KVLFF-functionalized MNPs has been experimentally demonstrated comparing the Aβ_1−42_ concentration in the fluid before and after being processed by the above-described device. It is a differential measurement: knowing the initial concentration of Aβ_1−42_ in the injected fluid and measuring it at the end of the experiment, the binding probability can be calculated as the difference. The initial concentration was measured in the standard solution before mixing it with MNPs at the beginning of the experiment. The supernatant fluid (in the tubing part before the cut) is then recovered to measure the concentration of the non-trapped Aβ_1−42_ at the end of the experiment. To this aim, the tube is cut by non-magnetic scissors just before the position of the magnets when they arrive to the final point and the supernatant fluid therein contained is analyzed.

To measure Aβ_1− 42_ concentrations we used a LUMIPULSE® G600II instrument (Fujirebio, Ghent, Belgium). It is a magnetic-bead-based immunoassay automated system accepted for clinical analysis of human CSF samples. To detect Aβ_1− 42_, it automatically adds, after a washing step, streptavidin-conjugated alkaline phosphatase (AP) to the biotinylated monoclonal antibody on the beads. Subsequently, those beads bind onto 3-(2′-spiroadamantane)-4-methoxy-4-(3ʺ-phosphoryloxy) phenyl-1, 2-dioxetane disodium salt (AMPPD) used as substrate. The luminescence at 477 nm is finally measured to give a quantitative estimate of the amyloid concentration.

**Fig. 7:**
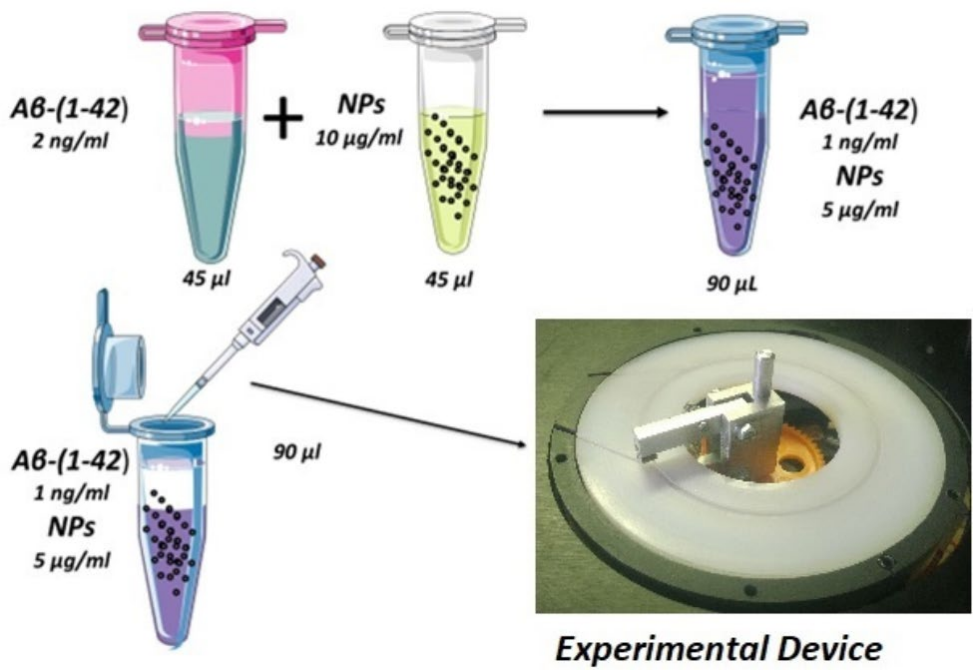
Sketch of the experimental procedure used to assess Aβ1-42-MNPs binding probability. Dilution is needed to match the fluid volume processable by Lumipulse® with the one contained in the experimental device. Numerical values of concentrations are given in the figure for the numerical ratio 1:1

An Aβ_1-42_ – MNP binding probability as high as 90% was obtained. This value was achieved at Aβ_1-42_ concentration of 1 ng/ml and MNP concentration of 5 µg/ml to have a 1:1 ratio between nanoparticles and amyloids (Table 1). Indeed, as the mass of one nanoparticle is 37‧10^–18^ g and the mass of one Αβ_1-42_ molecule is 7.47‧10^-21^ g) the ratio in terms of mass must be 5000:1 to have an equal number of MNPs and Αβ_1-42_ in the solution. For the sake of completeness, in Table 1 are shown also the raw experimental results of the measured concentration in each experiment. This is the value measured by Lumipulse® after applying the dilution factor as we need to match the fluid volume processable by Lumipulse® (~ 150 µl). The experimental procedure is sketched in Fig. 7.

**Table 1:** Experimental data on binding rate between KVLFF-functionalized MNPs and Aβ_1− 42_ molecules when they are mixed in 1:1 numerical ratio in the experimental device. Data refer to amyloid concentration, as measured by Lumipulse®, in the supernatant solution after the magnets completed two rotations to cover the two-loop spiral as detailed in the text. The binding ratio is calculated as the difference between the initial concentration and the one after the process

Aβ concentration (µg /ml)	MNP concentration (µg/ml)	Numerical ratio (Aβ/MNP)	Dilution factor	Initial Aβ concentration - $\varvec{A}\varvec{\beta }0$ - (pg/ml) *after dilution	Final Aβ concentration -$\varvec{A}\varvec{\beta }\varvec{f}$ - (pg/ml)	Binding rate (%) $1-\frac{\varvec{A}\varvec{\beta }\varvec{f}}{\varvec{A}\varvec{\beta }0}$
REAL CONCENTRATION			RAW EXPERIMENTAL RESULTS			
0.001	**5**	1:1	1/5	408	8	**98**
0.001	**5**	1:1	1/5	422	60	**86**
0.001	**5**	1:1	1/5	398	22	**94**
0.001	**5**	1:1	1/5	403	62	**85**

Binding ratio average: 90%

Control experiments have been performed with bare (non-aptamer-functionalized) MNPs, and no decrease in the Aβ_1-42_ concentration have been measured (Table 2) within the statistical variability in the Lumipulse® measurements valued in 50 pg/ml. It demonstrates the specific molecular binding between Aβ_1-42_ and RGen3K(KLVFF)_16_ branched aptamers chemically bonded onto the MNPs surface

**Table 2:** Experimental data on binding rate between non-functionalized MNPs and Aβ_1−42_ molecules when they are mixed in 1:1 numerical ratio. The experimental procedure behind is the same as for the data in Table 1. No decrease in the Aβ_1−42_ concentrations has been measured

Aβ concentration (ng/ml)	MNP concentration (µg/ml)	Numerical ratio (Aβ/MNP)	Dilution factor	Initial Aβ concentration - $\varvec{A}\varvec{\beta }0$ - (pg/ml) *after dilution	Final Aβ concentration -$\varvec{A}\varvec{\beta }\varvec{f}$ - (pg/ml)	Binding rate (%) $1-\frac{\varvec{A}\varvec{\beta }\varvec{f}}{\varvec{A}\varvec{\beta }0}$
REAL CONCENTRATION			RAW EXPERIMENTAL RESULTS			
1	**5**	1:1	1/5	366	384	**0**
1	**5**	1:1	1/5	338	365	**0**

Binding ratio average: 0%

Due to the tendency of Aβ_1−42_ to adhere onto plastic surfaces [12], unspecific adhesion of Aβ_1−42_ on the medical-grade silicone tube has been included in the analysis by injecting a Aβ_1−42_ solution into a silicone tube and let the solution static for 3.5 h (magnets running time). No measurable change of Aβ_1−42_ concentration has been measured (Table 3)

**Table 3:** Experimental data on unspecific adhesion of Aβ_1−42_ molecules on medical grade silicone. No decrease in the Aβ_1−42_ concentration has been measured. Note that the Aβ_1−42_ concentration used in this experiment is roughly 2/3 of the ones used in the experiments of Tables 1 and 2

Aβ concentration (pg/ml)	MNP concentration (µg/ml)	Numerical ratio (Aβ/MNP)	Dilution factor	Initial Aβ concentration - $\varvec{A}\varvec{\beta }0$ - (pg/ml) *after dilution	Final Aβ concentration -$\varvec{A}\varvec{\beta }\varvec{f}$ - (pg/ml)	Binding rate (%) $1-\frac{\varvec{A}\varvec{\beta }\varvec{f}}{\varvec{A}\varvec{\beta }0}$
REAL CONCENTRATION			RAW EXPERIMENTAL RESULTS			
579	**0**	//	1/6	75	87	**0**

Binding ratio average: 0%

In summary, the experimental procedure here described allows: (1) to maximize the binding probability of the MNPs with the Aβ_1−42_ molecules; and (2) to retrieve most (if not all) the Aβ_1−42_ molecules sparsely distributed within a large volume of fluid. Because of the low concentration, the binding probability would have been negligible if the Aβ_1−42_ molecules and the MNPs were left free to move in the solution by Brownian motion. Instead, in the device the MNPs are attracted by the magnets to form a plug with the CSC configuration optimized for Aβ_1−42_ capture. Also, the magnetic field generated by the magnets would not be able to cover the entire spiral - or, more generally, any macroscopic volume – but, moving the magnets along the spiral, allows them to collect the particles at any point when the magnets will pass by. Even though, some Aβ_1−42_ can be captured by randomly meeting free MNPs in the solution, the dominant process of molecular binding is by mean of the MNPs densely packed in the magnetically-actuated CSC plug. The working principle of the device thus allows, after the complete rotation, the concentration of virtually all (~ 90%) the Aβ_1−42_ molecules dispersed in a macroscopic volume of 100 µl into a dense plug of ca. 50 nl that, theoretically, can be analyzed by ultra-sensitive nanosensors. Also, larger volumes can be processed as more loops in the spiral design can be added to accommodate larger amount of analyte.

**Fig. 8:**
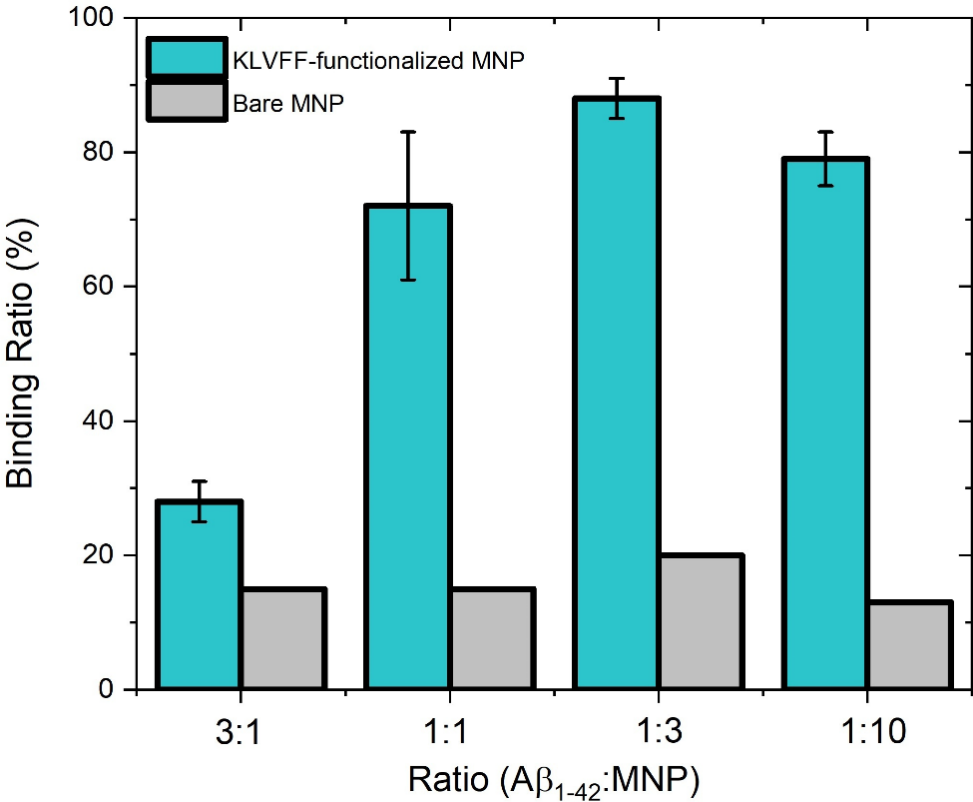
Quantitative analysis of the molecular binding between Αβ_1 − 42_ and KLVFF-functionalized MNPs as a function of the numerical ratio Αβ_1 − 42_: MNP (green histograms). As a control it is shown the binding ratio between bare MNPs (grey histograms). Aβ_1 − 42_ concentrations for the experiments with 1:1 and 1:3 ratios – 600 pg/ml and 200 pg/ml, respectively – are usually found in clinical samples. 600 pg/ml (1:1 ratio in the figure) in particular is considered the cut-off value for early AD diagnosis [39, 40]

A series of experiments has been performed by systematically varying the numerical ratio between MNPs and molecules. Four different ratios (Αβ _1-42_: MNP), were tested: 3:1; 1:1; 1:3 and 1:10. As shown in Fig. 8, with 1:1 or lower ratios, we obtained similar binding rates of around 80% whose difference lies within the experimental error. Indeed, using a one-way analysis of variance (ANOVA) followed by Tukey’s multiple comparison test (P < 0.05), no statistical difference was observed. Instead, a significantly lower value of ~ 30% was obtained with the 3:1 ratio. These results indicate that increasing the number of MNPs beyond the parity with the Αβ_1-42_ molecules does not significantly increase the binding efficiency whereas, when the Αβ_1-42_ molecules outnumber the MNPs, we observe a drastic decrease. Interestingly, the binding rate in the 3:1 experiment (where there are three molecules for each MNP) is consistent with the hypothesis where one MNP binds with just one molecule, as, in such case, just a third of the molecules can be captured. On the other hand, lowering the Αβ _1-42_: MNP ratio to 1:10 – thus in a condition where the MNPs outnumber the Αβ _1-42_ molecules by a factor of 10 – the binding rate remains constant. Taken together, these experimental results offer the tantalizing view of a one-to-one molecular binding where each nanoparticle binds with just one Aβ_1-42_ molecule. Because of the need to provide a volume of fluid suitable for the Lumipulse® analysis, we chose to use a dilution factor of 1/3 in this experimental series to have a homogeneous set of data. The decrease in the binding rate for the 1:1 ratio experiments with different dilution factors from 90% to 1/5 (Table 1) to 78% for 1/3 (Table 4) is apparent but it can be explained by the lower concentration of the Aβ_1-42_ molecules in the experiment with 1/3 dilution factor. Interestingly, the cubic root of the ratio between the concentrations used (600 pg/ml and 1000 pg/ml) – a quantity related with the ratio of the molecular free path – is very similar to the ratio of the binding rate with the respective concentrations. This hints that the decrease in the binding rate is due to the fact the molecules are more dispersed when using lower concentration and thus the probability for them to meet the MNP is also smaller. The technology here presented can thus achieve a control on molecular scale on capture, separation, and transport of proteins in macroscopic samples over macroscopic distances.

**Table 4:** Experimental data on binding rate between KVLFF-functionalized MNPs and Aβ_1−42_ molecules when they are mixed with different numerical ratios in the experimental device. The experimental procedure behind is the same as for the data in Table 1

Aβ concentration (pg/ml)	MNP concentration (µg/ml)	ratio (Aβ/MNP)	Dilution factor	Initial Aβ concentration - $\varvec{A}\varvec{\beta }0$ - (pg/ml) *after dilution	Final Aβ concentration -$\varvec{A}\varvec{\beta }\varvec{f}$ - (pg/ml)	Binding rate (%) $1-\frac{\varvec{A}\varvec{\beta }\varvec{f}}{\varvec{A}\varvec{\beta }0}$	Average
REAL CONCENTRATION			RAW EXPERIMENTAL RESULTS				
600	3	1:1	1/3	220	51	77	**78%**
600	3	1:1	1/3	231	47	80	
600	3	1:1	1/3	248	57	78	
600	3 (bare MNP)	1:1	1/3	202	172	15	**15%**
200	3	1:3	1/3	82	7	91	**88%**
200	3	1:3	1/3	61	7	89	
200	3	1:3	1/3	80	12	85	
200	3 (bare MNP)	1:3	1/3	67	54	20	**20%**
1800	3	3:1	1/3	544	384	30	**28%**
1800	3	3:1	1/3	546	409	25	
1800	3	3:1	1/3	529	363	31	
1800	3 (bare MNP)	3:1	1/3	476	407	15	**15%**
60	3	1:10	1/3	19	5	75	**79%**
60	3	1:10	1/3	17	3	83	
60	3	1:10	1/3	20	4	80	
60	3 (bare MNP)	1:10	1/3	16	14	13	**13%**

## Conclusion

Even though the treatment for late-stage AD remains a distant prospect, a first example of a drug able to slow cognitive decline in patients with early AD has been recently published [8]. This highlights the critical importance of an AD early diagnosis. Moreover, it is broadly accepted that many potential therapies are being used too late and only after consistent neuronal damage was established. Aβ species from CSF are widely recognized as an optimal biomarker for an early detection of the disease; yet, the utility of Aβ_1−42_ as a robust biomarker in clinics is still questioned due to technical challenges in its measurement [12, 38]. The development of automatized low-cost point-of-care (PoC) diagnostic tools can solve many clinical and analytical impediments and the device here presented has several innovative technological characteristics for this application; namely, (1) it does not require microfluidic pumps/switches or elaborated chip design; (2) the capture/binding process occurs in a reservoir of static fluid by magnetic nanoparticles that travels through the fluid actuated by external magnetic fields. This reduces the need of pumps or metering to control the flux of fluid in the apparatus as the pump is used only to fill the capillary at the beginning of the experiments; (3) the separation of Aβ_1−42_ molecules from the solution is done, with very high efficiency, without using any membrane; (4) the processes of separation, concentration and molecular transport is done simultaneously.

In this study a new technology has been developed to capture, separate, and manipulate a molecular biomarker, Aβ_1−42_, in a concentration range relevant for AD diagnosis in clinical practice: 500–1000 pg/ml [39, 40]. It is based on functionalized MNPs actuated by external magnetic fields and an efficiency of 90% in biomarker extraction has been experimentally demonstrated with clinically-relevant volumes of solution. Moreover, such efficiency can be easily improved via dedicated engineering. Owing the scalability of the magnetic fields, the technology can be smoothly adapted to various clinical settings and needs. Noteworthy, it is also compatible with the materials currently used in hospitals and medical laboratories. Still, in spite of the convincing demonstration performed in laboratory conditions, we are aware that further studies using real CSF samples form patients at different level of cognitive impairment are required for a clinical validation of this technology.

Finally, although demonstrated for Aβ_1−42_, the technology here presented is not restricted to this but can be applied to a variety of molecular biomarkers: for example, among the others, to monitor bone metastases in oncology [41] or to assess radiation injury [42]. It can also lay the foundations for versatile and ultrasensitive platforms for viral detection [43, 44].

## Data Availability

All data associated with this study are available in the main text.
